# Antiproliferative Activity of Flavonoids from *Croton sphaerogynus* Baill. (Euphorbiaceae)

**DOI:** 10.1155/2015/212809

**Published:** 2015-05-17

**Authors:** Kátia Pereira dos Santos, Lucimar B. Motta, Deborah Y. A. C. Santos, Maria L. F. Salatino, Antonio Salatino, Marcelo J. Pena Ferreira, João Henrique G. Lago, Ana Lúcia T. G. Ruiz, João E. de Carvalho, Cláudia M. Furlan

**Affiliations:** ^1^Departamento de Botânica, Instituto de Biociências, Universidade de São Paulo, Rua do Matão 277, 05508-090 São Paulo, SP, Brazil; ^2^Universidade Paulista, Rua Apeninos 267, 01533-000 São Paulo, SP, Brazil; ^3^Instituto de Ciências Ambientais, Químicas e Farmacêuticas, Universidade Federal de São Paulo, Rua Professor Arthur Riedel 275, 09972-270 Diadema, SP, Brazil; ^4^Divisão de Farmacologia e Toxicologia, CPQBA, CP 6171, Universidade Estadual de Campinas, 13083-970 Campinas, SP, Brazil

## Abstract

*Croton sphaerogynus* is a shrub from the Atlantic Rain Forest in southeastern Brazil. A lyophilized crude EtOH extract from leaves of *C. sphaerogynus*, obtained by maceration at room temperature (seven days), was suspended in methanol and partitioned with hexane. The purified MeOH phase was fractionated over Sephadex LH-20 yielding five fractions (**F1–F5**) containing flavonoids, as characterized by HPLC-DAD and HPLC-MS analyses. The antiproliferative activity of the crude EtOH extract, MeOH and hexane phases, and fractions **F1–F5** was evaluated on *in vitro* cell lines NCI-H460 (nonsmall cell lung), MCF-7 (breast cancer), and U251 (glioma). The MeOH phase showed activity (mean log GI_50_ 0.54) higher than the hexane phase and EtOH extract (mean log GI_50_ 1.13 and 1.19, resp.). **F1** exhibited activity against NCI-H460 (nonsmall cell lung) (GI_50_ 1.2 *μ*g/mL), which could be accounted for the presence of flavonoids and/or diterpenes. **F4** showed moderate activity (mean log GI_50_ 1.05), while **F5** showed weak activity (mean log GI_50_ 1.36). It is suggested that the antiproliferative activity of the crude EtOH extract and MeOH phase is accounted for a synergistic combination of flavonoids and diterpenes.

## 1. Introduction

*Croton* L. (Euphorbiaceae) has approximately 1300 species of herbaceous, shrubs, trees, and lianas forms. The genus is widely distributed in tropical and subtropical regions around the world, including Brazil, a country with 316 species, among which 253 are endemic [1, 2].

Medicinal and toxic properties of* Croton* species have been ascribed to a wide variety of chemical compounds, such as terpenoids and steroids, alkaloids, and phenolic compounds, the latter including predominantly flavonoids, lignans, and proanthocyanidins [3–5].

According to the International Agency for Research on Cancer (IARC), the world impact of cancer has more than duplicated in the last 30 years [6]. The 2014 and 2015 annual estimative regarding Brazil foresees the emergence of approximately 576,000 new cancer cases, including nonmelanoma skin cancer. This latter cancer type is expected to become the most frequent among the Brazilian population (32% new cases), followed by prostate tumors (12%), female breast (10%), colon and rectum (6%), lung (5%), stomach (3.5%), and cervical (3%) [7].

Among drugs currently used in cancer treatment, over 60% are products directly or indirectly derived from plants [8]; most of them related to alkaloids and terpenoids [9].

Several studies dealing with* Croton* species have reported the isolation of cytotoxic derivatives. Shoots of* C. hieronymi* Griseb. showed strong activity against lung A-549 carcinoma cells, mouse lymphoma, and human colon carcinoma [10]. The CH_2_Cl_2_ extract of leaves of* C. macrobothrys* Baill. and* C. zambesicus* Müll. Arg. showed cytotoxicity against human lung and leukemia cells [11] and cervix carcinoma cells [12], respectively. Ent-kaurane diterpenoids from* Croton tonkinensis* have activity against colorectal cancer cells [13];* ent*-kaur-16-ene-6a,19-diol from* C. floribundus* Spreng. exhibited a moderate effect against MDA-MB-435 (melanoma), HCT-8 (colorectal adenocarcinoma), and HCT-116 (colorectal adenocarcinoma) cell lines [14]. Isopimara-7,15-dien-3b-ol from* C. zambesicus* showed weak cytotoxic activity against cancer (HeLa, human cervix carcinoma cells; HL-60, human promyelocytic leukemia) and noncancer (WI-38, human lung fibroblast) cell lines [15]. An epimer of kaurenoic acid from* C. antisyphiliticus* Mart. showed cytotoxic activity, with half maximal inhibitory concentration values of 59.41, 68.18, and 60.30 *μ*g/mL for the B-16 (murine melanoma), HeLa (human cervix carcinoma cells), and 3T3 (normal mouse embryo fibroblasts) cell lines, respectively [16]. These compounds are considered valuable toward the development of new and highly effective anticancer chemotherapeutic agents, due to their efficacy toward the induction of apoptosis [17, 18].

Individuals of* Croton sphaerogynus* Baill. sect.* Cleodora* grow in the states of Bahia, Rio de Janeiro, Espírito Santo, and São Paulo (Brazil). Most of its populations are distributed in moist forests of seashore plains (“restinga forests”). Two other species of this section,* Croton cajucara* Benth., popularly known as “sacaca,” and* Croton heterocalyx* Baill., have been traditionally exploited for their medicinal properties (e.g., [4, 5, 19]). CH_2_Cl_2_ and hexane extracts from leaves of* C. sphaerogynus* exhibited antiproliferative activity against NCI-H460 (nonsmall cell lung) (GI_50_ 0.26 *μ*g/mL and 0.33 *μ*g/mL, resp.) and K562 cell lines (GI_50_ 0.60 *μ*g/mL and <0.25 *μ*g/mL, resp.). These activities were related to the presence of abietane, podocarpane, and clerodane diterpenes [20].

The main goal of this study was to characterize the major polar constituents of the EtOH extract from leaves of* C. sphaerogynus* and evaluate their* in vitro* antiproliferative activities against tumor cell lines. This study differs from that of Motta et al. [20], which focused on the antiproliferative activity of diterpenes of nonpolar extracts from* C. sphaerogynus* and thus did not deal with its flavonoid constituents and their antiproliferative activity.

## 2. Material and Methods

### 2.1. Plant Material

Leaves of* Croton sphaerogynus* were collected in January 2012, in an area of Atlantic Forest in the municipality of Itanhaém, State of São Paulo (Southeastern Brazil). A voucher specimen (LBM 65) was deposited in the herbarium Maria Eneyda P. K. Fidalgo (SP), São Paulo.

### 2.2. Extraction Procedure

Dried and powdered leaf material (1 kg) was extracted by maceration with EtOH (5 L) for seven days at room temperature. Crude EtOH extract (**EE**) was concentrated under reduced pressure, evaporated to dryness under a stream of nitrogen, and lyophilized, affording 70.62 g of crude EtOH extract (yield: 7%). Part of the crude EtOH extract (65 g) was resuspended in MeOH and partitioned using hexane. The hexane phase (**HP**) was concentrated under reduced pressure to yield 20 g (2%) of hexane phase, while the MeOH phase (**MP**) was lyophilized to afford 15 g (yield: 1.5%). Part of** MP** (8 g) was fractionated over Sephadex LH-20 using MeOH as eluent, affording five fractions:** F1 **(2 g),** F2** (0.119 g),** F3** (0.090 g),** F4** (0.353 g), and** F5** (0.090 g).

### 2.3. Chemical Analysis

All lyophilized crude** EtOH** extract,** MP** and** HP** phases, and fractions (**F1**–**F5**) were dissolved in MeOH (2 mg/mL) and analyzed using a HPLC 1260 (Agilent Technologies) chromatograph, equipped with diode array detector (DAD) and a Zorbax-C18 (250 × 4.6 mm, 3.5 *μ*m, Agilent, USA) column at 40°C. Solvents used were 0.1% acetic acid (AcOH) and acetonitrile (CH_3_CN), starting with 15% of CH_3_CN (0–20 min), increasing to 100% (20–25 min), isocratic (5 min), and decreasing to 15% (30–32 min), and isocratic (3 min). Solvent flow rates were 1.5 mL/min (0–25 min), 1.0 mL/min (25-26 min), and 1.5 mL/min (26–35 min); injection volume was 3 *μ*L, and detection was at 352 nm and 280 nm. Quercetin and kaempferol at concentrations from 0.6 to 360 ng/mL were used to prepare calibration curves following the same analysis conditions. Results are expressed in milligrams per gram of dry sample (mg/g).

MeOH phase (**MP**) was also analyzed using a Bruker Daltonics equipment Esquire 3000 Plus HPLC with a Zorbax-C18 (250 × 4.6 mm, 3.5 *μ*m, Agilent, USA) column at 40°C, using the same conditions cited above. Solvent flow rates was .90 *μ*L/min, voltage 4000 V, nebulizer 27 psi, drying gas at 320°C, and flow of 7 L/min. Constituents were identified by comparing the corresponding UV-Vis and ESI/MS-MS spectra with MS data from the literature.

A purified compound from** F5** was analyzed by ^1^H NMR at 500 MHz, using a Bruker DRX-500 spectrometer. DMSO-d_6_ (Aldrich) was used as solvent and the residual peak of the nondeuterated solvent as internal standard.

### 2.4. Antiproliferative Assay

Cancer cell lines used were kindly provided by the National Cancer Institute (NCI) at Frederick MA-USA and included NCI-H460 (nonsmall cell lung), MCF-7 (breast cancer), and U251 (glioma). Stock cell cultures were grown in medium containing RPMI 1640, supplemented with 5% of fetal bovine serum. Experimental cultures were supplemented also with penicilin : streptomicin (10 *μ*g/mL : 10 UI/mL).

Cells (100 *μ*L cells/well, inoculation density from 3–6 × 10^4^ cell/mL) in 96-well plates were exposed to various sample concentrations (0.25 to 250 *μ*g/mL, 100 *μ*L/well) in DMSO/RPMI 1640/FBS 5% at 37°C, 5% of CO_2_ in air for 48 h. Final DMSO concentration did not affect cell viability. Cells were then fixed with 50% trichloroacetic acid and cell proliferation was determined by spectrophotometric quantification of cellular protein content at 540 nm, using the sulforhodamine B assay. Doxorubicin (DOX; 0.025–25 *μ*g/mL) was used as positive control. Three measurements were obtained at the beginning of incubation (time zero, *T*_0_) and 48 h after incubation for compound-free (*C*) and tested (*T*) cells. Cell proliferation was determined according to the equation 100 × [(*T* − *T*_0_)/*C* − *T*_0_], for *T*_0_ < *T* ≤ *C*, and 100 × [(*T* − *T*_0_)/*T*_0_], for *T* ≤ *T*_0_ and a concentration-response curve for each cell line was plotted using software ORIGIN 7.5 (OriginLab Corporation) [21].

### 2.5. Data Analysis

Using the concentration-response curve for each cell line, GI_50_ (concentration causing 50% growth inhibition) [22] was determined by means of nonlinear regression analysis, using software ORIGIN 7.5 (OriginLab Corporation). The average activity (mean of log GI_50_) of the extracts tested was also determined using MS Excel software. Extracts were regarded as inactive (mean > 1.5), weakly (1.1 < mean < 1.5), moderately (0 < mean < 1.1), or potently (mean < 0) active on basis of the NCI criteria for the mean of log GI_50_ [23].

## 3. Results and Discussion

Retention times and UV and MS data analysis of the constituents from the crude EtOH extract (**EE**), MeOH (**MP**) and hexane (**HP**) partition phases, and fractions** F1**–**F5** are given in Table 1. Twenty substances were detected, mainly quercetin and kaempferol derivatives.** F1, F2,** and** F3** exhibited similar flavonol composition.** F4** contains three major compounds: quercitrin, quercetin 3-*O*-methyl ether, and a kaempferol derivative, while** F5** contains quercetin 3-*O*-methyl ether (Table 1, compound** 17**).

Comparison of spectroscopic data with those from the literature [24] allowed the identification of compound** 17** as 5,7,3′,4′-tetrahydroxy-3-methoxy-flavonol (quercetin-3-*O*-methyl ether) (Table 1).

Flavonoids found in* Croton* are mostly highly methoxylated aglycones, such as artemetin [3]. It has been reported that several* Croton* species produce acylated flavonoids, such as tiliroside (kaempferol-*p*-coumaroyl glucoside) [25–27]. Rutin (Table 1, compound** 7**) was also detected in the MeOH extract from leaves of* C. lechleri* Mull. Arg. [28]; fresh latex of* C. celtidifolius* Baill. has flavonols, such as kaempferol, quercetin, and myricetin in its composition [29]. Savietto et al. [30] detected apigenin dihexoside and tiliroside (kaempferol-*p*-coumaroyl glucoside) as ubiquitous constituents of the MeOH leaf extract of* C. dichrous* Müll. Arg.,* C. erythroxyloides* Baill., and* C. myrianthus* Mull. Arg.

*C. sphaerogynus* was previously described as a major producer of diterpenes. Using serial extraction with hexane, CH_2_Cl_2_, and MeOH, Motta et al. [20] identified a great diversity of diterpenes, especially in the CH_2_Cl_2_ extract. In the present study, maceration with EtOH at room temperature yielded an extract with a diterpene profile similar to that described by Motta et al. [20]. The lyophilized crude ethanol extract was resuspended in MeOH and partitioned with hexane. Partition did not eliminate the diterpenes from the polar fraction** MP**. Column chromatography using Sephadex and MeOH gave five fractions (**F1**–**F5**), the first of which (**F1**) contained diterpenes and flavonoids, while the further fractions (**F2**–**F5**) contained flavonoids exclusively.

Crude EtOH extract(**EE**), hexane phase (**HP**),** F1**, and** F2** showed weak antiproliferative activity (Table 2). Methanol phase (**MP**) and** F4** showed moderate activity, mainly against cell lines NCI-H460 (nonsmall cell lung) (mean log GI_50_ 0.54 and 1.05, resp.). On the other hand,** F3** was inactive, while** F5**, containing virtually only quercetin-3-*O*-methyl ether (Table 1, compound** 17**), also showed weak antiproliferative activity (Table 2).

According to Motta et al. [20] the antiproliferative activity of* C. sphaerogynus* extract was a result of the massive presence of abietane and/or podocarpane diterpenes in nonpolar extracts. The present study tested two different sets of samples: extract and phases composed by different proportions of diterpenes and flavonoids (**EE**,** HP**,** MP**,and fraction** F1**) and fractions composed exclusively by flavonoids (**F2**–**F5**).  Table 3 compares data obtained by Motta et al. [20] and the diterpene mixture obtained in the present work. According to Motta et al. [20] the CH_2_Cl_2_ extract showed higher activity (mean log GI_50_ 0.86), compared with hexane and MeOH extracts (mean log GI_50_ 1.26 and 1.49, resp.). Regarding the diterpene profile, the latter extract was the most similar to** MP** and** HP** phases, although some qualitative and quantitative differences are evident. No crotonin derivative was detected in the present study, which may explain the moderate antiproliferative activity of the CH_2_CL_2_ extract (mean 0.86 log GI_50_) reported by Motta et al. [20] and the weak antiproliferative activity exerted by** HP**. The CH_2_Cl_2_ extract from* C. macrobothrys*, which contains a crotonin derivative, showed moderate antiproliferative activity (mean log GI_50_ 0.89) [11]. Grynberg et al. [31] tested* trans*-dehydrocrotonin and* trans*-crotonin isolated from* C. cajucara* Benth. on the survival of mice bearing Sarcoma 180 and Ehrlich carcinoma and observed a significant antitumor activity when mice were treated with* trans*-dehydrocrotonin.

**MP** phase was the most active sample, showing moderate antiproliferative activity. The relative proportion of diterpenes and flavonoids (Table 4) might be important to enhance the antiproliferative activity. Either extracts with high contents of diterpenes or fractions with high contents of flavonoids presented weak or no antiproliferative activity.** F2**,** F3**, and** F4** (fractions lacking diterpenes) were shown to be inactive.** F1**, though still containing diterpenes, showed flavonols in smaller amount than** MP**;** F5**, composed virtually by quercetin-3-*O*-methyl ether, also showed weak antiproliferative activity. The hexane phase (**HP**) contained no detectable flavonoids. These results suggest that* Croton* species lacking crotonin derivatives might have moderate antiproliferative activity if they have a combination of other diterpenes and flavonols.

Extracts from* Croton* species are frequently reported as exerting antiproliferative activity. The essential oil from the stem bark of* C. lechleri *showed mutagen-protective efficacy [32] and crude extracts from stems of* C. cajucara* [19], containing clerodane diterpenes, exert antitumor activity against the K562 leukemic cell line. The CH_2_Cl_2_ extract of* C. macrobothrys* leaves, also containing clerodane diterpenes, exhibited moderate antiproliferative activity against several cell lines, in particular NCI-H460 (nonsmall cell lung) and K562 (leukemia) [11].

On the other hand, flavonoids such as apigenin dihexoside and tiliroside (kaempferol-*p*-coumaroyl glucoside) detected in leaf extract of* C. dichrous, C. myrianthus*, and* C. erythroxyloides* showed weak or no growth cell inhibition. However, extracts or fractions with substantial amounts of these compounds showed weak activity [30]. However, MeOH extract from* C. erythroxyloides* obtained under reflux showed moderate antiproliferative activity (mean of log GI_50_ 1.00) [30].

Angst et al. [33] observed that the flavonol quercetin inhibits the growth of pancreatic cancer cell lines by inducing apoptosis. The association of gemcitabine (a standard chemotherapeutic drug administered to patients with pancreatic cancer) and quercetin had no additional effect when compared with quercetin administered alone. The authors also observed a significant apoptotic effect and reduced tumor cell proliferation in* in vivo* assay using quercetin.

Besides synergism, the chemical structure of the flavonoids seems to be directly related to their antiproliferative activity. Burmistrova et al. [34] showed that synthetic flavonols with a hydroxyl group at the C3 position are 7-fold more potent than flavonols with a methoxyl group at the same position. This result suggests that a C3 methoxyl group at C3 is a reducing factor of cytotoxicity. In the present study, quercetin-3-*O*-methyl ether alone showed weak antiproliferative activity (mean of log GI_50_ 1.36). However, according to Seito et al. [35] flavonoids with methoxyl groups at positions other than C3 seem to have inhibitory effect on cell growth.

## 4. Conclusion

The antiproliferative activity of* Croton sphaerogynus* seems to be related to the presence of diterpenes and flavonoids. MeOH phase (**MP)** presented the highest antiproliferative activity among all samples tested and is showed to be composed by diterpenes and a high amount of flavonoids, in comparison with the crude EtOH extract (**EE**) and** F1**. Fractions containing no diterpenes showed weak antiproliferative activity. Samples containing small proportions of flavonoids also showed weak antiproliferative activity. The relative proportions of representatives of these two metabolite classes (flavonoids and diterpenes) in* C. sphaerogynus* extracts seem to be crucial to determine their antiproliferative activity.

## Figures and Tables

**Table 1:** Major constituents of the crude EtOH extract (**EE**), MeOH (**MP**) and hexane (**HP**) phases, and fractions (**F1–F5**) from of *Croton sphaerogynus* leaves, characterized by HPLC-DAD (352 nm) and HPLC-MS.

Constituent	Rt (min)	UV/visible (nm)	Mass spectrum		Suggestion
			[*m*/*z*]^+^	[*m*/*z*]^−^	
**1**	1.75	254, 264 (om), 348			Quercetin derivative
**2**	2.96	256, 264 (om), 296 (om), 354	757.8; 611.1; 465; 303	755.7	Quercetin triglycoside (2 rhamnose; 1 hexose)
**3**	3.16	260, 296, 356	741.8; 595.1; 433.1; 286.8		Kaempferol triglycoside (2 rhamnose; 1 hexose)
**4**	4.05	264, 292, 346	741.8; 595.1; 287		Kaempferol triglycoside (2 rhamnose; 1 hexose)
**5**	4.30	264, 294, 348	741.9; 595.2; 448.7; 287	739.6	Kaempferol triglycoside (2 rhamnose; 1 hexose)
**6**	4.99	256, 266 (om), 294 (om), 354	595, 303		Quercetin diglycoside (1 pentose; 1 hexose)
**7**	5.33	256, 266 (om), 294 (om), 354	661.1; 465	608.9	Rutin
**8**	6.04	264, 294, 348	595, 286.9	593	Kaempferol diglycoside (1 rhamnose; 1 glucose)
**9**	6.35	256, 264 (om), 296 (om), 354	479, 316.9		Isorhamnetin monoglycoside (1 hexose)
**10**	7.30	264, 294, 348	448.9; 286.9		Kaempferol monoglycoside (1 hexose)
**11**	9.23	264, 294, 346			Kaempferol derivative
**12**	10.10	256, 268 (om), 356			Quercetin derivative
**13**	10.72	264, 294, 346			Kaempferol derivative
**14**	10.97	256, 264 (om), 306 (om), 348	449.0; 302.9	447	Quercitrin
**15**	20.44	262, 294, 340			Kaempferol derivative
**16**	22.95	254, 270, 298, 370			Quercetin
17^*^	23.23	256, 266 (om), 298 (om), 356	317, 302	314.8	Quercetin 3-*O*-methyl ether
**18**	23.55	264, 292, 320, 366			Kaempferol
**19**	23.73	266, 294, 350			Kaempferol derivative
**20**	23.84	254, 268 (om), 294 (om), 356			Quercetin derivative

Rt: average retention time in minutes. ^*^Compound confirmed by ^1^H NMR.

**Table 2:** Antiproliferative activity (GI_50_, *µ*g/mL) of the leaf extract and fractions of *Croton sphaerogynus* on culture cell lines.

Material tested	Cell lines^a^			Mean log GI_50_^c^
	U251	MCF-7	NCI-H460	
Doxorubicin^b^	<0.025	<0.025	<0.025	−1.60 **P**
**EE**	23.4	53.4	3.00	1.19 W
**MP**	8.10	20.0	0.25	0.54 **M**
**HP**	24.7	27.2	3.80	1.14 W
**F1**	16.7	20.3	1.20	1.19 W
**F2**	41.8	61.1	12.3	1.50 W
**F3**	58.7	47.0	12.4	1.51 I
**F4**	15.0	8.10	11.8	1.05 **M**
**F5**	31.6	17.9	21.8	1.36 W

^a^U251: glioma; MCF-7: breast cancer; NCI-H460: nonsmall cell lung.

^b^Positive control. NCI's criteria (Fouche et al., 2008 [23]): I: mean log GI_50_ > 1.5 = inactive; W: weak activity: mean log GI_50_ > 1.1–1.5; M: moderate activity: mean log GI_50_ > 0–1.1; P: potent activity: mean log GI_50_ < 0.

^c^log GI_50_.

Crude EtOH extract (**EE)**; MeOH** (MP**) and hexane **(HP)** phases, and fractions (**F1**, **F2**, **F3**, **F4**, and **F5**).

**Table 3:** Main constituents of leaf extract and fractions from *Croton sphaerogynus* leaves and respective GC/EIMS data. **EE**: crude EtOH extract; **MP**: methanol phase; **HP**: hexane phase; **F1**: fraction 1. RT: retention time (min); MM: molecular mass; N.I.: not identified.

	RT (min)	MM	Characterization^*^	Relative amount (%)					
				Dch^*^	Met^*^	EE	MP	HP	F1
1	14.8	272	Abieta-8, 11-diene	2.2	2.3	—	—	3.8	—
2	15.2	278	NI			—	0.9	1.6	—
3	16.2	306	NI			—	—	1.3	—
4	16.4	286	Abieta-8,11-dien-3-one	20.2	20.1	36.0	22.3	27.6	23.8
5	16.7	286	Abieta-8,11,13-trien-3-ol	6.2	13.1	5.4	6.5	7.7	7.1
6	16.9	288	Abieta-8,11-dien-3-ol	3.8	16.7	4.6	8.0	3.3	8.9
7	17.2	286	Podocarp-7-en,13-methyl-13-vinyl-3-one	12.9	4.7	11.2	17.9	18.5	17.9
8	17.5	286	NI			2.1	2.5	0.9	2.7
9	17.7	288	NI			0.9	3.8	1.5	4.6
10	18.0	284	NI			3.6	1.7	1.6	1.4
11	18.1	286	NI			—	1.3	1.8	1.6
12	18.2	286	NI			6.7	4.0	3.3	3.4
13	18.6	286	Abieta-8,11,13-trien-12-ol	11.9	18.5	10.3	13.9	21.8	13.5
14	19.2	288	Podocarp-7-en-3-ol, 13-methyl-13-vinyl	4.5		1.6	6.2	4.0	6.0
15	19.3	302	13-hydroxy-abieta-8,11-dien-7-one	4.8		4.2	2.1	—	1.9
16	20.3	300	11-hydroxy-abieta-8,11,13-trien-7-one	3.4		2.2	0.9	—	1.2
17	20.5	302	NI			—	1.2	—	1.3
18	20.6	300	Abieta-6,8,11,13-tetraen-3,12-diol	0.7		10.2	4.8	0.7	4.2
19	21.1	286	NI			—	1.2	—	0.4
20	21.2	302	Abieta-8,11,13-trien-6,14-diol	0.8		0.9	0.9	—	—
21	—	316	Crotonin derivative^*^	5.2		—	—	—	—

NI				23.4	24.6	8.8	9.7	7.1	7.8

^*^Main constituents of leaf extract of *Croton sphaerogynus* and respective GC/EIMS data and characterization according to Motta et al. [20]. Dch, CH_2_Cl_2_; Met, MeOH.

**Table 4:** Relative amount of major flavonoids in crude EtOH extract (**EE**), MeOH phase (**MP**), and fractions (**F1–F5**) of *C. sphaerogynus*, detected by HPLC-DAD (352 nm).

Constituent^*^	**EE**	**MP**	**F1**	**F2**	**F3**	**F4**	**F5**
**1**	nd	0.10^Q^	—	0.47^Q^	—	—	—
**2**	0.20^Q^	1.17^Q^	0.94^Q^	—	—	—	—
**3**	nd	—	—	2.23^K^	3.00^K^	—	—
**4**	0.57^K^	2.45^K^	2.01^K^	—	—	—	—
**5**	1.03^K^	3.73^K^	3.04^K^	—	—	—	—
**6**	0.09^Q^	0.35^Q^	—	4.65^Q^	5.18^Q^	—	—
**7**	0.37^Q^	1.63^Q^	0.89^Q^	19.35^Q^	8.39^Q^	—	—
**8**	0.10^K^	0.51^K^	0.50^K^	—	—	—	—
**9**	0.09^Q^	0.48^Q^	—	0.91^Q^	6.51^Q^	8.42^Q^	—
**10**	0.18^K^	0.67^K^	0.48^K^	5.5^K^	1.84^K^	—	—
**11**	0.99^K^	3.04^K^	2.42^K^	19.71^K^	4.42^K^	—	—
**12**	nd	—	—	7.47^Q^	8.39^Q^	—	—
**13**	nd	—	—	4.15^K^	—	—	—
**14**	0.51^Q^	3.13^Q^	—	3.65^Q^	34.51^Q^	47.06^Q^	—
**15**	nd	—	—	2.54^K^	7.89^K^	—	—
**16**	nd	—	—	2.47^Q^	3.68^Q^	nd	—
**17**	0.59^Q^	3.26^Q^	0.13^Q^	3.06^Q^	2.16^Q^	70.96^Q^	58.31^Q^
**18**	nd	—	—	2.94^K^	1.70^K^	—	—
**19**	0.48^K^	0.97^K^	—	—	2.13^K^	29.68^K^	—
**20**	nd	—	—	—	2.23^Q^	nd	—

^*^Identification suggestions in Table 1.

^Q^Values expressed as milligram per gram of quercetin equivalent (mg/g QE).

^K^Values expressed as milligram per gram of kaempferol equivalent (mg/g KE).

nd: trace amounts.
